# 多发性骨髓瘤遗传学检测中国专家共识（2026年版）

**DOI:** 10.3760/cma.j.cn121090-20250925-00442

**Published:** 2026-01

**Authors:** 中国临床肿瘤学会（CSCO）骨髓瘤专家委员会 中国抗癌协会（CACA）血液肿瘤专业委员会骨髓瘤与浆细胞疾病学组

## Abstract

遗传学异常是多发性骨髓瘤（MM）预后评估的关键指标，也是现代风险分层体系的重要基础。临床应规范开展染色体核型分析、荧光原位杂交（FISH）及二代测序（NGS）等检测，以识别高危患者并指导个体化治疗。随着全基因组测序、单细胞测序等技术的发展，对MM克隆演化与遗传异质性的认识不断加深，而三药、四药联合治疗的应用亦改变了部分遗传学异常的预后意义，提示现有风险体系需更新。为规范我国MM遗传学检测实践，中国临床肿瘤学会（CSCO）骨髓瘤专家委员会与中国抗癌协会（CACA）血液肿瘤专业委员会骨髓瘤与浆细胞疾病学组基于最新证据对2019年版共识进行了修订。本版重点包括：完善检测流程、统一高危遗传学异常判读与报告规范，并探讨新型分子标志物在风险分层中的应用。新版共识将促进我国MM诊疗的规范化与精准化，提升对高危患者的识别与管理能力。

## Abstract (en)

Genetic abnormalities are a cornerstone for prognostic assessment in multiple myeloma（MM）and form the basis of contemporary risk‐stratification systems. In routine clinical practice, cytogenetic karyotyping, fluorescence in situ hybridization, and next‐generation sequencing should be performed in a standardized manner to identify high‐risk patients and guide individualized therapy. With recent advances in genomic technologies, such as whole-genome sequencing and single-cell sequencing, our understanding of clonal evolution and genetic heterogeneity in MM has deepened. Meanwhile, the use of triplet and quadruplet combination regimens has altered the prognostic impact of certain genetic lesions, highlighting the need to update the existing risk frameworks. To standardize genetic testing for MM in China, the Multiple Myeloma Expert Committee of the Chinese Society of Clinical Oncology（CSCO）and Myeloma & Plasma Cell Disease Group, Hematology Oncology Committee of the China Anti-Cancer Association（CACA）have revised the 2019 consensus based on the latest evidence. This updated consensus focuses on refining the testing workflows, harmonizing the interpretation and reporting of high-risk genetic abnormalities, and discussing the incorporation of emerging molecular markers into risk stratification. This new consensus aims to promote standardized and precision-based MM care in China and improve the identification and management of high-risk patients.

多发性骨髓瘤（multiple myeloma, MM）是一种以克隆性浆细胞异常增殖为特征的血液系统恶性肿瘤，MM患者的生存期存在显著异质性。建立基于预后因素的风险分层体系对于指导个体化治疗具有重要意义。遗传学异常是MM预后评估的核心指标，通过精准检测识别高危患者亚群已成为MM诊疗的重要组成部分。然而，目前国内临床中心在遗传学检测技术的应用及结果解读方面仍缺乏统一规范。与此同时，随着三药及四药联合治疗方案的广泛应用，遗传学异常的预后价值亦呈现出新的变化[1]。鉴于此，中国临床肿瘤学会（CSCO）骨髓瘤专家委员会联合中国抗癌协会（CACA）血液肿瘤专业委员会骨髓瘤与浆细胞疾病学组组织专家基于最新循证医学证据对2019年版共识进行了系统修订。本次修订旨在优化遗传学检测流程，规范高危遗传学异常的判读标准与报告模式，并探索将新型分子标志物整合至现有风险分层体系，以进一步推动我国MM诊疗的规范化与精准化。

一、MM遗传学检测技术的选择

MM的遗传学检测体系主要包括传统染色体核型分析、荧光原位杂交（fluorescence in situ hybridization, FISH）、染色体微阵列分析（chromosomal microarray analysis, CMA）以及二代测序（next-generation sequencing, NGS）等技术。上述方法均可识别MM相关遗传学异常，各具优势与局限。临床实践中，应合理选择并联合应用多种检测手段，以更全面和精准地评估MM遗传学异常，为风险分层和个体化治疗提供有力依据[2]。

（一）染色体核型分析

染色体核型分析常用方法包括G显带、R显带等。该技术通过观察中期分裂象检测MM患者的染色体数目和结构异常，如超二倍体（约见于50％的初诊患者）、亚二倍体、14q32染色体易位、染色体扩增、缺失及复杂核型异常等。然而，由于MM肿瘤细胞整体增殖率较低，且部分患者骨髓标本中肿瘤细胞比例不足，传统制备方法往往难以获得足够的分裂象，导致染色体异常的检出率偏低，限制了核型分析在MM中的临床应用。

（二）间期FISH（interphase FISH, iFISH）

FISH利用荧光标记探针与特定DNA序列杂交，可有效检测染色体易位、缺失及扩增等遗传学异常。其中，iFISH因不依赖细胞分裂象，特别适用于增殖活性低的MM细胞，已成为MM遗传学检测的临床金标准。iFISH可在单细胞水平识别微小结构异常，具有较高的灵敏度和特异性，其异常检出率明显优于传统核型分析。该方法对样本要求较低，操作简便，结果重复性良好，结合浆细胞纯化技术，还可进一步提升准确性，因此被国际指南广泛推荐为MM遗传学评估的常规方法，亦适用于多中心研究与临床实践。然而，FISH检测仅针对特定遗传学异常，难以全面评估患者的整体遗传学风险。此外，当前不同实验室在探针组合、检测流程及结果判读方面仍存在差异，亟需进一步规范和统一，以提升其在临床应用中的一致性和准确性。

（三）NGS

MM的NGS技术主要包括全基因组测序（whole genome sequencing, WGS）、全外显子组测序（whole exome sequencing, WES）、全转录组测序（whole transcriptome sequencing, WTS）以及靶向基因测序（targeted gene sequencing, TGS）。其中，WGS因成本高、分析复杂，目前多用于基础研究；WES受限于测序深度，临床应用有限；WTS可用于构建基因表达谱（如GEP70、SKY92），虽具预后价值，但不同模型间差异较大，限制了临床推广。相比之下，TGS因成本相对较低、检测范围明确，可有效检测KRAS、NRAS、TP53、BRAF等关键基因突变，并兼顾拷贝数变异（copy number variation, CNV）的评估。在这些分子事件中，TP53突变与不良预后密切相关，具有相对稳定的独立预测价值，因此MM的风险评估与分层建议常规采用NGS检测TP53基因突变。目前除TP53外，其余基因突变的独立预后价值有限。

（四）其他检测技术

CMA不依赖中期分裂象，可在全基因组范围内检测CNV。常用方法包括单核苷酸多态性微阵列（SNP-A）和微阵列比较基因组杂交（aCGH）。其中，SNP-A不仅可检测拷贝数异常，还能识别单亲二倍体（UPD）及中性杂合性丢失（CN-LOH），被认为是CNV检测的金标准，广泛用于血液学肿瘤。其可发现：①整条染色体数目异常；②片段缺失或扩增，最小至100 kb；③复杂结构异常；④UPD/CN-LOH，但其无法识别涉及IgH的平衡易位，因此联合FISH可将MM遗传学异常的检出率提高至95％以上；⑤对于初诊MM（NDMM）或复发难治性MM（RRMM），SNP-A联合FISH可以作为患者遗传学异常检测的一线方法，也可以根据患者的经济状况和标本情况，将SNP-A技术作为二线检测方法。当SNP-A作为二线检测方法时，可以为以下场景提供重要信息：①根据患者的常规FISH结果进行遗传学预后分层后，患者的临床表现和疗效不符合分层预期时，SNP-A检测可以提供患者的额外遗传学信息；②常规FISH检测结果为阴性时，SNP-A结果可以提供更全面的遗传学背景；③基于肿瘤浆细胞的异质性，患者复发时，FISH可能未检测到或仅检测到部分初诊时的异常，SNP-A结果对于解释患者复发时的遗传学异常状况非常有必要；④当FISH结果出现多个信号，尤其是1q21探针出现4、6甚至8个信号，但无法判断是浆细胞的DNA整体的倍体增加（四倍体常见）导致，还是浆细胞DNA其实为二倍体，仅探针所及区域的拷贝数增加，这时SNP-A的检测非常重要，有助于鉴别肿瘤浆细胞的倍体和真正的遗传学异常状态。光学基因组图谱（optical genome mapping, OGM）通过在长链DNA特定位点荧光标记并成像，可获得全基因组结构变异图谱。其灵敏度高，可检测约500 bp以上的变异，准确定位易位断点、受累基因及CNV范围。研究显示，OGM与FISH结果一致性达98％～100％，且能识别部分FISH遗漏的异常。但其对样本要求高、受限于成本和通量，目前临床应用有限。数字定量PCR（digital PCR, dPCR），尤其是微滴式数字PCR（ddPCR），可在无需标准品的情况下实现靶标分子绝对定量，能区分微小浓度差异，检测灵敏度、精密度及重复性高，且操作简便，是值得探索的MM遗传学新检测手段。

**专家共识**：①检测技术选择原则：推荐综合应用染色体核型分析、CMA、FISH及NGS等方法，以提高MM遗传学异常的检出率与准确性。②染色体核型分析：可覆盖全基因组，检测染色体数目及结构异常（如超二倍体、低二倍体、易位、缺失及复杂核型等），但受制于MM细胞增殖率低，异常检出率有限。③iFISH：已成为MM遗传学检测的临床金标准，可在单细胞水平精准识别多种特定异常。但其检测范围局限于预设探针，且不同实验室在探针组合及结果判读方面存在差异，亟需进一步规范与统一。④CMA：可在全基因组范围检测染色体数目异常、结构异常及约100 kb以上的小片段扩增或缺失（如超二倍体、低二倍体、易位、缺失、复杂核型等）。其中，SNP-A还可检测UPD和CN-LOH。但CMA无法识别平衡易位，因此临床应联合FISH，以提升检出率。⑤NGS：TGS可有效检测关键基因突变（如KRAS、NRAS、TP53、BRAF）及CNV。在已知分子事件中，TP53突变与不良预后密切相关，具有独立预测价值，建议常规采用NGS检测TP53基因突变，用于风险评估与分层，其他单基因突变的独立预后意义尚需进一步验证。

二、MM的遗传学检测流程

（一）取材及样本处理

MM遗传学检测常面临肿瘤浆细胞比例偏低、分布不均及骨髓抽吸过程中外周血稀释等问题，导致检测准确性受限，因此常规进行浆细胞富集至关重要。临床常用方法包括CD138免疫磁珠分选和流式细胞术分选，其中前者因设备要求低、操作相对简便而被广泛应用，通常10～20 ml骨髓标本即可满足检测需求，但其存在耗时和成本较高等不足。部分研究提出，在FISH检测中联合胞质轻链免疫荧光（cIg-FISH），通过轻链特异性抗体标记可更精准地识别异常浆细胞，但因抗体选择性、成本及显微镜通道限制，临床应用尚受限。对于灵敏度较高的NGS检测，在特定情境下可适当降低富集要求，但总体仍推荐常规进行浆细胞富集以确保结果的可靠性。标本处理方面，FISH及核型分析宜使用肝素钠抗凝，而cIg-FISH、NGS及SNP-A则推荐乙二胺四乙酸（EDTA）或枸橼酸钠作为抗凝剂；标本应尽早送检，建议24 h内完成浆细胞富集和制片，最长不超过72 h（4 °C保存，超时CD138抗原可能从细胞表面脱落，导致CD138磁珠单抗富集浆细胞失败或富集效率低）。

**专家共识**：①骨髓标本采集要点：推荐使用肝素钠、EDTA或枸橼酸钠作为抗凝剂，以避免细胞凝集及形态改变。标本采集后应尽快送检，理想情况下应在24 h内完成浆细胞富集与制片，最长不超过72 h；若超时，应经实验室验证细胞稳定性后方可检测。②浆细胞富集方法及纯度要求：为确保检测准确性，推荐常规进行浆细胞富集。常用方法包括CD138免疫磁珠分选和流式细胞术分选，其中优先推荐CD138免疫磁珠分选，因其操作简便且临床可行性高。对于NGS检测，在部分特定情境下可适当降低浆细胞纯度要求，但总体上仍建议常规进行浆细胞富集。

（二）检测靶点选择

MM遗传学异常常见且复杂。t（4;14）约见于15％的NDMM患者，导致FGFR3和（或）NSD2过表达；t（14;16）及t（14;20）分别见于3％～5％和1％～2％的NDMM，分别导致MAF和MAFB的过表达。这些IGH易位常伴随继发性遗传学异常、较高的突变负荷及APOBEC活性增强。t（11;14）可见于15％～20％的NDMM及约50％原发性浆细胞白血病，导致CCND1过表达，该类患者对BCL2抑制剂可能更敏感。TP53缺失在NDMM中发生率为7％～10％，在RRMM及继发性浆细胞白血病中发生率明显升高；双等位基因失活（双缺失或缺失/CN-LOH合并突变）预示更差预后。1p缺失在8％～12％的NDMM中可见，以1p32.3区（CDKN2C、FAF1）最具代表性；1q21获得/扩增是MM常见高危因素，在NDMM、RRMM中的发生率分别为30％～45％、55％～70％。检测推荐使用CKS1B探针，并联合1p探针以提高准确性。MYC重排（8q24.21）可导致MYC表达升高，见于30％～45％的NDMM和约50％的RRMM，常伴其他遗传学异常，与疾病进展密切相关。尽管尚未普遍纳入风险分层模型，2024年版美国国家综合癌症网络（National Comprehensive Cancer Network，NCCN）指南已将MYC重排列为高危异常。

目前，MM的FISH检测靶点的选择仍存在一定争议，临床实践中建议参考国际权威指南及最新循证医学证据。根据2025年版NCCN指南（V2），推荐在NDMM患者中常规检测以下异常：del（13q）、del（17p13）、t（4;14）、t（11;14）、t（14;16）、t（14;20）、1q21获得/扩增及1p32缺失，并将MYC重排列入高危遗传学异常。实验室可先采用IGH断裂探针进行初筛，若发现异常（包括IGH的5′或3′端缺失），则进一步检测t（4;14）、t（14;16）、t（14;20）和t（11;14）。此流程既可降低检测成本，又能合理利用有限的浆细胞样本。

目前，MM的NGS检测靶点尚无统一标准。鉴于TP53基因突变预后意义明确，推荐常规检测TP53突变，其他靶点选择建议根据临床需求和实验室条件进行定制化设计。基因选择应综合考虑预后价值、靶向潜力、耐药预测和突变频率，具备条件的实验室可进一步开展CNV和UPD/CN-LOH检测。TP53的结构包括转录激活域、DNA结合域（外显子5～8）和C端调控域，突变多集中于DNA结合区，常见热点为R273、R248、G244、R175、R280等，类型以错义突变为主，无义和移码突变亦可存在。建议对TP53（17p13.1）全部11个外显子及上下游10 bp剪接区进行检测；如条件受限，至少覆盖外显子5～8，可识别约85％的致病性变异。目前，MM尚缺乏统一的基因表达检测靶点，国内研究者提出了一种七基因特征评分，并将其与国际分期系统（ISS）相结合，形成了MR-ISS模型。该模型可显著提高风险分层能力，且已建立了基于ddPCR技术的检测体系。但其临床应用仍需通过多中心前瞻性研究验证[3]。

**专家共识**：①FISH检测靶点：建议参考2025年NCCN指南（V2），推荐检测的关键异常包括：del（13）、del（17p13）、t（4;14）、t（11;14）、t（14;16）、t（14;20）、1q21获得/扩增、del（1p32）及MYC重排。TP53双等位基因失活、1p32缺失（CDKN2C区域）和1q21扩增（CKS1B）应联合检测，以提高风险评估的准确性。②NGS检测靶点：目前尚无统一标准，推荐常规检测TP53突变。TP53突变主要集中于DNA结合结构域（外显子5～8），建议检测覆盖全部外显子及剪接区，以提高致病性变异识别率。

（三）阳性阈值

阳性阈值的界定是MM遗传学检测中最具争议的问题之一，目前尚无统一标准，不同研究机构差异显著。在FISH检测中，实验室常以10～20例正常骨髓细胞的检测结果为参照，采用“*x*+3*SD*”或95％*CI*的逆贝塔函数设定阳性阈值。但由于MM的遗传学检测多基于浆细胞富集，实验室阈值并不能完全反映临床意义，因此需区分“实验室阳性阈值”和“临床阳性阈值”。由实验室参照正常对照和公式计算出来的阳性阈值是对正常人群出现阳性细胞真实上限的估计，定义为实验室阳性阈值；根据患者的临床资料进行统计学分析得出的对患者疗效和预后有影响的最低阳性细胞比例定义为临床阳性阈值。实验室阳性值仅提示存在一定比例的异常浆细胞，而是否具有临床意义应参考国际指南推荐的对临床有影响的阈值标准。在具体异常中，IGH易位为原发性异常，其检出率较稳定，阈值设定对诊断影响有限；而del（17p）、1q21获得/扩增等继发性异常随疾病进展而增加，阈值设定对于灵敏度与准确性尤为关键。欧洲骨髓瘤工作组（European Myeloma Network, EMN）将FISH中融合或断裂探针的临床阈值设为10％，数目异常探针的临床阈值为20％[4]。此外，del（17p）的肿瘤克隆比例（cancer clonal fraction, CCF）与预后密切相关[5]–[6]，检测报告中应明确标注，以辅助风险评估。由于探针、试剂、平台及标准操作流程（standard operating procedure，SOP）差异，不同实验室需建立各自的阈值标准。

在MM相关基因的NGS检测中，目前同样缺乏统一阈值。阈值设定应综合检测目的、平台性能、变异类型及临床相关性评估。一般推荐在测序深度≥1 000×时，以等位基因变异频率（variant allele frequency，VAF）≥5％作为阳性阈值；对于部分明确的致病热点突变（如TP53 R273H），若技术准确性可靠，可将阈值降低至1％～2％，前提条件包括：①目标位点测序深度≥500×；②突变同时获正反向读段支持（每方向≥2条，且突变位于读段中部）；③突变位点不处于高噪音区域（如同聚物区域）。对于低VAF突变，应在质量控制达标的前提下谨慎报告。

**专家共识**：①FISH检测阈值：目前尚无统一标准，建议各实验室结合自身条件建立适用的阈值。至少计数100个细胞。IGH易位可采用相对宽松的判定标准（如10％）；而对于随疾病进展频率升高的继发性异常［如del（17p）、1q21获得/扩增］，应采取更严格、保守的阈值判定，并在报告中注明克隆比例，以辅助风险评估。②NGS检测阈值：在平均测序深度≥1 000×时，常见突变类型建议采用VAF≥5％作为阳性阈值；对于明确的致病热点突变，在特定技术条件下阈值可下调至1％～2％。

（四）检测时间点

克隆演变是MM疾病进展和治疗耐药的核心机制，常伴随继发性遗传学异常的出现，以复发阶段最为显著。因此，建议在MM复发时常规重新评估遗传学特征。高危异常如del（17p）、TP53突变及1q21获得/扩增的克隆扩展，与疾病复发和耐药密切相关[7]。2025年版NCCN指南（V1）已将复发时新出现的此类异常定义为“动态高危”预后因素，强调动态监测遗传学变化在个体化治疗决策和预后评估中的重要价值。

**专家共识**：①检测时机与项目：推荐在MM患者初诊及每次复发时常规开展遗传学检测。复发患者至少应重新评估del（17p）、TP53突变及1q21获得/扩增及del（1p）；若初诊时检测项目不全，可参照NDMM的推荐检测靶点。②动态高危因素：MM患者在复发时若新出现del（17p）和（或）TP53突变、1q21获得/扩增，应列为“动态高危”预后因素，用于指导风险分层和个体化治疗决策。

（五）FISH结果解读及报告

FISH检测结果应按照最新版《国际人类细胞遗传学命名系统》（International System for Human Cytogenomic Nomenclature，ISCN）进行标准化书写，并辅以规范术语以提升报告的可读性和临床解释性。结果解读应包含以下关键要素：明确所用探针名称及设计类型（如双色双融合探针、断裂探针），描述典型阳性与阴性信号模式；说明浆细胞富集方法（如CD138免疫磁珠分选、胞质轻链标记）、计数细胞总数（建议不少于100个）、异常信号细胞比例及采用的阳性阈值。对于基因缺失，应分别报告目标基因缺失与染色体单体缺失的比例；1q21异常需明确标注拷贝数，若出现非目标信号，应结合临床背景记录和解释。报告还应包含后续检测建议，以辅助临床决策。在四倍体或近四倍体背景下解读结果时需格外谨慎。例如，四倍体状态下若检测到TP53存在两个拷贝，应结合是否存在伴随TP53缺失的二倍体克隆共同判断；若染色体倍性评估受限，应避免使用“相对缺失”表述，并应提示TP53功能状态不确定，建议联合评估TP53突变。在近四倍体情况下，如观察到1q21六个拷贝，若同时存在明确二倍体克隆并显示1q21增加，更宜解释为“获得”而非“扩增”。

**专家共识**：FISH检测结果应遵循最新版ISCN命名系统，并采用标准化术语进行报告，需明确探针类型、浆细胞富集方法、阳性阈值及异常细胞比例。报告时应特别关注四倍体背景下1q21扩增及TP53缺失的解读，避免使用“相对缺失”等模糊表述，并建议结合TP53突变检测结果进行综合评估。

（六）NGS结果解读及报告

NGS靶向基因检测报告应遵循人类基因变异学会（HGVS）命名体系规范书写基因变异，并依据美国分子病理学协会（AMP）/美国临床肿瘤学会（ASCO）/美国病理学家学会（CAP）指南对变异进行致病性分级。同时，应对每个突变基因及其突变位点进行临床意义解读。报告中需包含患者及样本信息、测序深度、靶点列表、质控信息、专业术语解释及检测局限性说明，并应避免报告已知良性多态性位点（如TP53 P72R），以减少临床误判。根据国际骨髓瘤学会（IMS）和国际骨髓瘤工作组（IMWG）风险分层指南，TP53突变被列为高危因素，约6％的NDMM患者可检测到该突变，且常与TP53缺失同时存在；无论TP53突变还是缺失，单独存在即提示高危特征，因此建议同时进行TP53缺失与突变检测。检测样本宜为富集浆细胞后的骨髓标本，若使用未富集样本，则需谨慎解读阴性结果。

**专家共识**：①NGS靶向基因检测报告：MM患者的NGS靶向基因检测报告应包含完整的患者及样本信息、检测方法及其局限性说明，并遵循HGVS命名体系和AMP/ASCO/CAP指南进行标准化变异描述与致病性分级。所有变异需结合临床背景进行意义解读，同时应避免报告已知良性多态性变异。②TP53检测要求：TP53突变被IMS/IMWG等多项国际指南明确列为高危因素，建议在浆细胞富集的骨髓样本中进行检测；如使用未富集样本，对阴性结果需谨慎解读。

三、遗传学异常和MM的预后分层

细胞遗传学异常是MM风险分层的核心指标，但高危细胞遗传学异常（HRCA）的定义仍存在争议，并随研究进展不断演化。2016年IMWG指南将t（4;14）、t（14;16）、t（14;20）、del（17p）、1q21获得/扩增、亚二倍体核型、染色体核型del（13）及高危基因表达谱（GEP）纳入HRCA[8]。在此基础上，2025年版NCCN指南（V1）进一步增加了del（1p32）、TP53突变及MYC易位，并强调多重HRCA共存提示预后更差[9]。mSMART 3.0指南则将t（4;14）、t（14;16）、t（14;20）、del（17p）、TP53突变、1q21获得/扩增、del（1p）及高危GEP归入HRCA，同时提出“双打击”和“三打击”MM的概念，即存在≥2种或≥3种HRCA[10]。主要指南均一致认为TP53缺失和（或）突变为高度预后不良事件。

尽管MM治疗的进步改善了部分携带单一高危异常患者的结局，但合并多重HRCA的超高危MM仍预后极差。MASTER研究显示，即便采用微小残留病指导下的四药诱导治疗联合自体造血干细胞移植，也难以克服其不良生物学特征[11]。在四药治疗背景下，为统一高危MM（HRMM）的定义，IMS与IMWG提出了基因组分期共识（Consensus Genomic Staging, CGS），将以下情况界定为HRMM：①del（17p）克隆比例>20％和（或）合并TP53突变；②IGH易位［t（4;14）、t（14;16）、t（14;20）］合并1q+和（或）del（1p32）；③del（1p32）合并1q+或双等位基因缺失；④β_2_微球蛋白≥5.5 mg/L且肾功能正常[1]。该模型更强调遗传学异常共存模式对预后的影响。mSMART 4.0对高危异常的界定基本与CGS保持一致。

目前，细胞遗传学异常已被整合至多个风险分层体系，包括R-ISS、R2-ISS、mSMART 4.0、多参数异常评分系统（MASS）及NCCN预后模型，并作为核心组成部分。然而，鉴于MM显著的生物学异质性，现有模型仍无法完全精准识别所有高危患者。未来风险分层体系有待结合分子检测进展及治疗深度等进一步优化。

**专家共识**：①细胞遗传学异常与高危定义：细胞遗传学异常是MM风险分层的核心因素。各大指南均将t（4;14）、t（14;16）、t（14;20）、del（17p）、1q21获得/扩增等视为高危异常，但HRCA的定义仍在不断演化。多项指南（如NCCN 2025、mSMART 3.0/4.0）已将TP53突变、del（1p32）、MYC易位等纳入HRCA，并强调多重HRCA共存提示预后极差。②CGS的提出：IMS/IMWG提出的CGS进一步更新了HRMM的定义，突出遗传学异常组合模式对预后的决定性作用。③未来发展方向：未来风险分层体系需结合分子分型与治疗反应的动态监测，不断优化，以实现更精准的高危患者识别与管理。

四、总结

本次修订重点围绕MM遗传学检测的临床价值、技术规范及解读标准进行更新。首先，我们明确了遗传学异常在MM风险分层中的核心地位，推荐综合应用染色体核型分析、FISH、CMA及NGS等多种技术提高高危异常的检出率与准确性。其次，本共识对浆细胞富集的重要性、检测阈值的设定及报告格式提出了更严格的规范，确保结果的科学性与可比性。在HRCA的定义方面，结合最新循证证据，进一步纳入了TP53突变、del（1p32）及MYC易位，并强调多重HRCA共存提示预后极差。同时，本共识引入IMWG/IMS提出的CGS，突出遗传学异常组合模式的临床意义。总体而言，本次修订旨在推动我国MM遗传学检测的标准化与精准化，为临床风险评估和个体化治疗提供更加清晰、可操作的指导框架。
